# Synthesis, Photochemical and In Vitro Cytotoxic Evaluation of New Iodinated Aminosquaraines as Potential Sensitizers for Photodynamic Therapy

**DOI:** 10.3390/molecules24050863

**Published:** 2019-02-28

**Authors:** Filipa Mandim, Vânia C. Graça, Ricardo C. Calhelha, Isabel L. F. Machado, Luis F. V. Ferreira, Isabel C. F. R. Ferreira, Paulo F. Santos

**Affiliations:** 1 Centro de Química-Vila Real (CQ-VR), Universidade de Trás-os-Montes e Alto Douro, 5001-801 Vila Real, Portugal; filipamandim@ipb.pt (F.M.); vcssg_22@hotmail.com (V.C.G.); 2 Centro de Investigação de Montanha (CIMO), Instituto Politécnico de Bragança, Campus de Santa Apolónia, 5300-253 Bragança, Portugal; calhelha@ipb.pt; 3 CQFM-Centro de Química-Física Molecular and IN-Institute for Nanosciences and Nanotechnologies and IBB-Institute for Bioengineering and Biosciences, Instituto Superior Técnico, Universidade de Lisboa, 1049-001 Lisboa, Portugal; ilferreiramachado@tecnico.ulisboa.pt (I.L.F.M.); LuisFilipeVF@ist.utl.pt (L.F.V.F.); 4 Instituto Politécnico de Portalegre, P-7300-110 Portalegre, Portugal

**Keywords:** squaraine, singlet oxygen, photodynamic therapy

## Abstract

In this work, several benzothiazole-based aminosquaraine dyes, displaying strong absorption within the so-called phototherapeutic window (650–800 nm), were synthesized. The ability, of all the new dyes, to generate singlet oxygen was assessed by determining the correspondent phosphorescence emission and through the comparison with a standard. The quantum yields of singlet oxygen generation were determined and exhibited to be strongly dependent on the nature of the amino substituents introduced in the squaric ring. The photodynamic activity of the synthesized dyes was tested against four human tumor cell lines: breast (MCF-7), lung (NCI-H460), cervical (HeLa) and hepatocellular (HepG2) carcinomas; and a non-tumor porcine liver primary cell culture (PLP2). All the compounds synthesized were found to be able to inhibit tumor cells growth upon irradiation more than in the dark, in most of the cases, very significantly. Considering the photodynamic activity exhibited and the low toxicity displayed for the non-tumor cells, some of the synthetized dyes can be regarded as potential candidates as photosensitizers for PDT.

## 1. Introduction

Photodynamic therapy (PDT) has emerged as a very promising treatment for certain types of cancer and other non-oncologic disorders [1]. Over the years, PDT has received regulatory approval worldwide for several conditions and has now become an established therapeutic modality [2].

The basic principle underlying this therapeutic approach is simple: a light-absorbing molecule, after being administrated to the patient, is activated with light of an appropriate wavelength and gives rise to the production of reactive oxygen species (ROS). This species are responsible to induce damage of the surrounding cells [3]. Following interaction with light, the sensitizer, which is firstly excited to its excited singlet state, may efficiently populate, via intersystem crossing, the longer-lived excited triplet state. The triplet state of the sensitizer can then produce ROS by two different competing mechanisms [4]. In a Type I reaction, the sensitizer in the triplet state can react with organic substrates or solvents to generate free radicals that subsequently interact with oxygen to generate ROS such as superoxide radical anion and hydroxyl radical. Alternatively, it can transfer its energy directly to surrounding ground-state triplet oxygen to produce highly cytotoxic singlet state oxygen in a process called Type II reaction.

Both Type I and Type II reactions can occur in parallel. Their relative contribution depending chiefly on the nature of the sensitizer and the concentration of oxygen [5]. However, singlet oxygen, which acts catalytically, is generally considered to be the main cytotoxic agent responsible for the photodynamic effect [6]. Independently of the nature of the ROS generated during the photodynamic process, the ultimate outcome is the cell destruction, which can occur by three main mechanisms: direct cell death, either by apoptosis, necrosis or autophagy [7], vascular collapse and ensuing tissue hypoxia [8], and activation of inflammatory and immune responses [9]. Because the photodynamic process only takes place when the photosensitizer, oxygen and light are present simultaneously, PDT comes to be a particularly selective modality, with unique advantages compared to conventional tumor therapies such as surgery, chemotherapy and radiotherapy [10,11].

Photofrin^®^, a mixture of hematoporphyrin derivatives, was the first photosensitizer to receive regulatory approval in several countries for the treatment of various cancers. After more than twenty years is still the most frequently used PDT sensitizer in clinical practice [12]. Despite its popularity, Photofrin^®^ has several disadvantages, including chemical inhomogeneity, prolonged skin photosensitivity, low absorption of light and poor tissue penetration of light [13]. These drawbacks impelled a large research effort towards the development of new molecules that could meet the main requirements of an ideal sensitizer. Amongst these are [14]: (i) to be a single chemical entity; (ii) minimal dark toxicity and cytotoxicity only upon illumination; (iii) rapid clearance from the body to provide low systemic toxicity; (iv) high triplet quantum yield to efficiently generate ROS, namely single oxygen, upon irradiation; (v) strong absorption in the phototherapeutic window (650–800 nm), where the depth of light penetration in tissue is maximal, to allow the use of low drug dosages.

While no ideal photosensitizer has yet been discovered, a number of the so-called second generation photosensitizers have been developed, either porphyrin-based or non-porphyrin compounds, a small number of which have already granted regulatory approval for clinical oncotherapy while some of others are currently under clinical trials [12,15,16,17].

Squaraine dyes, which are the condensation products of squaric acid with electron rich substrates, are a family of compounds which have found wide application in the domain of photonics due to their unique properties, namely sharp and intense absorption within the visible to near-infrared range [18]. These same properties also turned them attractive for several biological applications, including as sensitizers for PDT [19]. As a consequence, structural modification of squaraine dyes become a very active area of research. Though a considerable number of different squaraine compounds has been developed envisioning their use as PDT sensitizers, both the in vitro and in vivo evaluation of their photodynamic capacity has been poorly addressed [20,21,22,23,24,25,26,27,28,29,30,31,32,33,34].

Aminosquaraines, a particular class of cationic squaraine dyes bearing an amino group replacing one of the oxygens atoms of the central four member ring, have recently been shown to possess photodynamic activity against several human tumor cell lines [35,36]. Aminosquaraines are envisioned to possess some potential advantages over the common zwitterionic counterparts as their cationic character may facilitate cellular uptake, benefiting from the cell’s membrane potential, while, at the same time, the amino group may conveniently favor the interaction with the biological medium. Moreover, besides the electron-donating character of the amino auxchromes red-shifts dye’s absorption [37,38], the presence of the amino group on the squaric ring may imparts a certain degree of additional rigidification to the molecule’s structure. This can diminish the non-radiative decay by photoisomerization leading to an inherent increase of the intersystem crossing efficiency from the singlet to the triplet state of the sensitizer, and, ultimately, of the efficiency of singlet oxygen generation.

Herein we describe the synthesis of several new symmetrical diiodinated aminosquaraines and the evaluation of their singlet oxygen generation ability and photocytotoxicity against selected human tumor cell lines.

## 2. Results and Discussion

### 2.1. Synthesis of Dyes

The molecular design rationale of the aminosquaraines synthesized was driven by the attempt to amend molecule’s rigidity, which influences the singlet oxygen production ability, dye’s maximum absorption, which depends on the electron-donating characteristics of the amine auxochrome, and the ability to establish intermolecular hydrogen bonds, which may increase water solubility and provide a potential means for interaction with biological substrates. Therefore, the strategy consisted in selecting substituent groups able to generate secondary and tertiary amines, some of which possessing flexible hydroxyethyl arms.

**Scheme 1:**
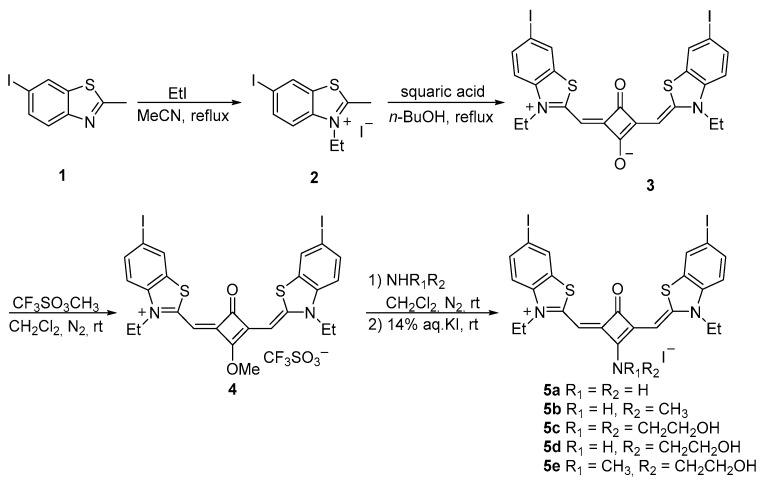
Synthesis of squaraine dyes **5a**–**e**.

Aminosquaraines **5a**–**e** were prepared by an expeditious synthetic method developed earlier by some of us [37], based on the methylation of one of the oxygen atoms of the squaraine core followed by nucleophilic substitution of the so-formed methoxy group by appropriate amines (Scheme 1).

Thus, condensation of two molar equivalents of benzothiazolium salt **2**, easily prepared by *N*-alkylation of 6-iodo-2-methylbenzothiazole (**1**) with iodoethane, with one equivalent of squaric acid in refluxing *n*-butanol/pyridine, resulted in the starting zwitterionic squaraine **3**. Methylation of the latter with methyl trifluoromethanessulfonate produced the crucial *O*-methyl ether derivative **4** from which the triflate analogues of **5a**–**e** could be obtained by treatment with the appropriate amines. Counter-ion exchange by iodine by treatment of the methanolic solutions of the dyes with excess 14% aqueous KI, produced the final aminosquaraine iodides **5a**–**e** in rather good yields. The counter ion exchange by iodine is carried out to potentially increase singlet oxygen generation through the external heavy atom effect [39]. As previously observed for several aminosquaraine dyes bearing a secondary amino group in the squaric ring [37,38], the two methylene protons of the polymethylene chain of **5b** and **5d** appear in the ^1^H-NMR spectrum as separated signals as consequence of the consequent local magnetic field inhomogeneity, resulting from the intramolecular hydrogen bonding and/or hindered rotation around the C-N bond linking the amino substituent to the squaric ring.

### 2.2. Photochemical Characterization

#### 2.2.1. UV-Visible Absorption Spectroscopy

**Figure 1:**
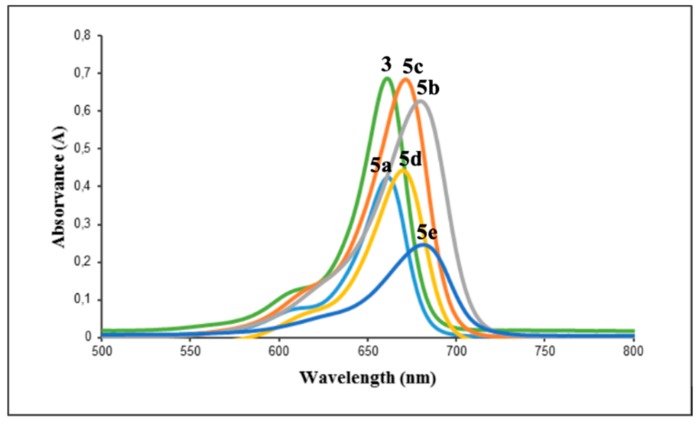
Absorption spectra of unsubstituted squaraine **3** and aminosquaraines **5a**–**e** in MeOH/CH_2_Cl_2_, 99/1 (*v*/*v*).

All synthesized aminosquaraines **5a**–**e** display sharp and strong absorption in the red end of the visible spectrum, within the phototherapeutic window (Figure 1, Table 1). The absorption of dyes **5a**–**e** is shifted bathochromically in relation to the non-substituted zwitterionic squaraine precursor **3**. The observed shifts ranging from 1 to 22 nm. The extent of the bathochromic shifts depends, in a direct way, on the electron donating ability of the amino auxochrome. This typical donor-acceptor mechanism is an usual feature of the chromophoric system of this class of compounds [37,38,40].

#### 2.2.2. Singlet Oxygen Quantum Yields

**Figure 2:**
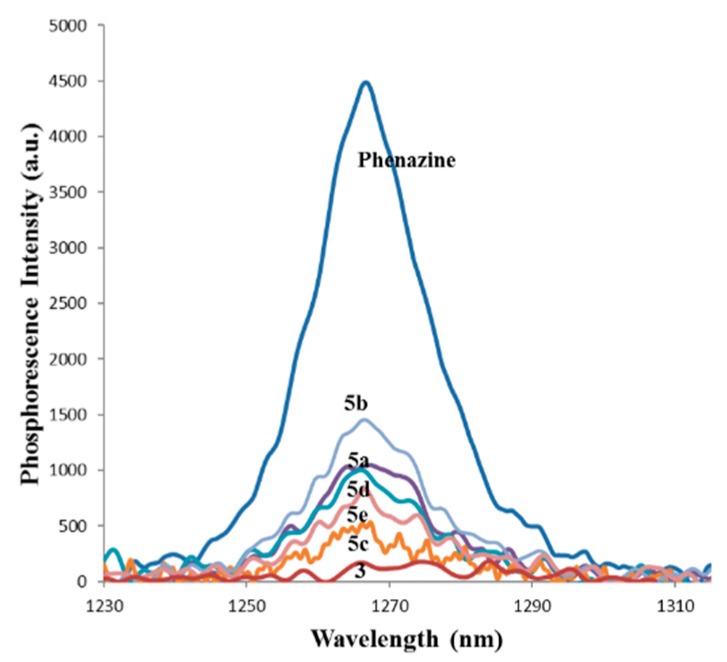
Singlet oxygen emission spectra of unsubstituted squaraine **3** and aminosquaraines **5a**–**e** in CHCl_3_ against the reference compound (phenazine).

The evaluation of the photosensitizing efficiency of the aminosquaraine dyes was performed in chloroform, by determining the singlet oxygen formation quantum yields (Φ_Δ_). Phenazine was used as standard [41,42]. The phosphorescence emission of singlet oxygen generated by the sensitizers at approximately 1270 nm is presented in Figure 2 and the determined quantum yields are summarized in Table 1.

These Φ_Δ_ values were obtained by a simple comparison of the integrated areas determined for the standard and samples, using the same OD at the excitation wavelength. For each determination an average of 100 phosphorescence spectra were used. Dye **5b** displays the highest quantum yield when compared to the other squaraine derivatives (Φ_Δ_ = 0.26). As previously reported [43,44], the amino group in the squaric ring enhances singlet oxygen generation efficiency when compared with the unsubstituted zwitterionic dye **3**. The results for Φ_Δ_ are strongly dependent on the nature of the amino substituents: the highest Φ_Δ_ values occurred for the smallest substituents, and the lowest values were observed for the hydroxyethylamino substituents, especially the bis(hydroxyethyl)-amino group (dye **5c**). This is related with the intramolecular charge transfer (ICT) mechanism which provides a non-radiative deactivation pathway, therefore decreasing the singlet oxygen formation quantum yields ([44] and references therein).

#### 2.2.3. Fluorescence Quantum Yields

Table 1 also presents the fluorescence quantum yields Φ_F_ for the aminosquaraines **5a**–**e** in CHCl_3_. As expected, due to the covalent link between iodine and the aminosquaraine, a strong heavy atom effect can be observed in all cases. This effect is emphasized in the case of the bis(hydroxyethyl)amino substituent group (dye **5c**).

### 2.3. In Vitro Photodynamic Activity

Four human tumor cell lines were used to assess the in vitro cytotoxicity of the synthesized dyes (**5a**–**e**): cervical (HeLa), breast (MCF7), hepatocellular (HepG2) and non-small cell lung (NCI-H460) carcinomas.

Although cytotoxicity was found to depend on the cell line, in general, all aminosquaraine dyes exhibited much higher cytotoxicity upon irradiation than in the absence of light. This fact revealed that the dyes are endowed with photodynamic action capacity. The assurance that the photodynamic effect was due, exclusively, to the sensitizing properties of the squaraine dyes, was secured by observing, in independent assays submitted to the photodynamic treatment and maintained in the dark, that, in the absence of the dyes neither DMSO nor light individually, nor the combination of both, were able to induce toxicity on the cells.

**Figure 3:**
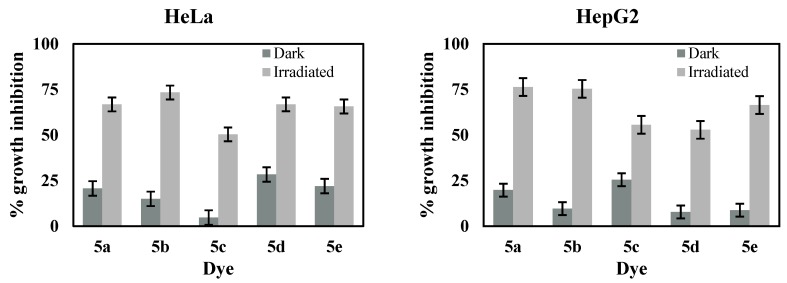

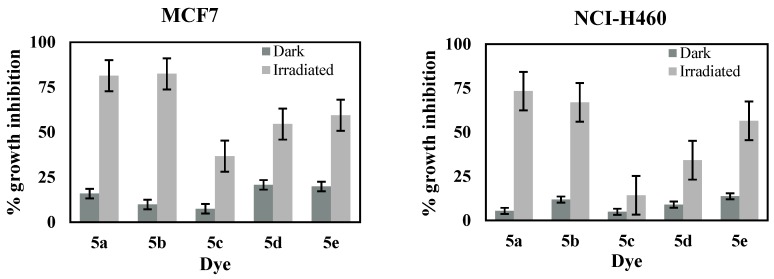
Growth inhibition of HeLa, HepG2, MCF-7 and NCI-H460 cell lines upon treatment with dyes **5a**–**e** (0.3 μM) in the dark and after irradiation. Results correspond to two independent assays and are presented as mean values ± standard deviation (SD).

Figure 3 shows the growth inhibition ability of aminosquaraines **5a**–**e** against the different human tumor cell lines, in the absence of light and upon irradiation, at a dye concentration of 0.3 μM. In this representative example, the growth inhibition in the dark was less than 20% in more than 75% of the assays.

Whatever the cell line, dyes **5a** and **5b** exhibited, invariably, the larger differences between the growth inhibition percentages upon illumination and in the dark (~ 46–68% and ~ 43–73%, respectively). In case of dye **5a** the more pronounced differences in growth inhibition under the two conditions was found against the MCF-7 cell line, closely followed by the NCI-H460 cell line, while for dye **5b** the larger difference was observed for the MCF-7 cell line. Dye **5c** displayed the weaker photocytotoxic capability, presenting the smaller differences of growth inhibition in the presence and in the absence of light, for the HepG2, MCF-7 and NCI-H460 cell lines. For the HeLa cells dye **5d** showed to be the less efficient sensitizer. It is worthwhile to mention that, though determined in CHCl_3_, dye **5c** exhibited the lowest singlet oxygen generation quantum yield among all dyes tested.

The GI_50_ values (concentration causing 50% cell growth inhibition) for aminosquaraines **5a**-**e** were also determined and are presented in Table 2. The differences found between the values determined for the assays in the dark and upon irradiation, clearly demonstrated once more the photocytotoxic ability of the dyes.

Among all compounds, **5a** and **5b** consistently presented the lowest GI_50_ values upon illumination for all the cell lines tested, ranging from 0.004 μM to 0.19 μM. In the absence of light the GI_50_ values increased ca. 80–1500 times for **5a** and ca. 45–250 times for **5b**, depending on the cell line. With exception of the HeLa cells, dye **5d** showed to be inactive in the dark (GI_50_ > 25 μM) against all the cells lines within the range of the concentrations used.

One of the desirable features of any new sensitizer is the lack of toxicity for non-tumor cells. For that reason aminosquaraines **5a**–**e** were also tested against a non-tumor porcine liver primary cell culture (PLP2) established by some of us, both in the absence of light and under irradiation. Dye **5a** presented a GI_50_ value under illumination considerably superior to those determined for the tumor cell lines. Dyes **5b**–**d** displayed GI_50_ values against PLP2 cells, both in the dark and under irradiation, not appreciably different than those obtained for the tumor cell lines used.

Having recently demonstrated the photocytotoxic properties of several aminosquaraine dyes derived from benzothiazole [36], we anticipated that the introduction of iodine atoms in the terminal aromatic nucleus of the dyes could increase singlet oxygen generation through the heavy atom effect and, ultimately, enhance their photocytotoxicity. In fact, the GI_50_ values obtained for dyes **5a**–**e** under irradiation were in general smaller, in some cases substantially, than those determined previously for their non-halogenated analogues. The GI_50_ values in the dark, on the other hand, were higher, making aminosquaraines **5a**–**e** safer for application.

## 3. Materials and Methods

### 3.1. Chemistry

#### 3.1.1. General Information

All reagents were obtained commercially and used as received. Solvents were of analytical grade. Anhydrous solvents were dried [45] and freshly distilled. All reactions were monitored by TLC using 0.20 mm aluminum-backed silica-gel plates (SIL G UV_254_, Macherey-Nagel, Düren, Germany). Melting points were measured in a melting-point apparatus equipped with a binocular microscope (Rotoquímica, Maia, Portugal) and are uncorrected. IR spectra were recorded on a Unicam Research Series FT-IR spectrophotometer (Mattson Instruments Inc., Madison, WI, USA); ν_max_ in cm^−1^. Vis spectra were performed on a Lambda 25 instrument (Perkin-Elmer, Singapore); λ_max_ in nm. ^1^H- (400.13 MHz) and ^13^C-NMR spectra (100.61 MHz) were recorded on an ARX 400 spectrometer (Brüker, Germany); δ in ppm relative to residual solvent signals, *J* in Hz. High resolution electrospray ionization time-of-flight mass spectra (HRMS ESI-TOF) were determined on Apex-Q FT-ICR and micrOTOF mass spectrometers (Bruker, Germany). 6-Iodo-2-methylbenzothiazole (**1**) was prepared as previously described [40].

#### 3.1.2. Synthesis of 3-Ethyl-6-iodo-2-methylbenzothiazolium iodide (**2**)

A mixture of 6-iodo-2-methylbenzothiazole (**1**, 6.00 g, 22.00 mmol) and a 3-fold excess of iodoethane (5.40 mL, 66.00 mmol) in CH_3_CN (150 mL) was heated under reflux for approximately 9 days. Upon cooling Et_2_O was added and the resulting precipitate was collected by filtration under reduced pressure and washed with Et_2_O. Yield: 68%. Yellowish crystals. M.p. > 265 °C (dec.).

#### 3.1.3. Synthesis of 4-[(3-Ethyl-6-iodobenzothiazol-3-ium-2-yl)methylidene]-2-[(3-ethyl-6-iodo-3*H*-benzothiazol-2-ylidene)methyl]-3-oxocyclobut-1-en-1-olate (**3**)

A mixture of benzothiazolium salt **2** (2.00 g, 4.64 mmol) and squaric acid (0.27 g, 2.32 mmol) in *n*-BuOH/pyridine [9/1 (*v*/*v*)] (230 mL) was heated under reflux for about 4 h. The reaction mixture was then cooled in an ice bath, the resulting precipitate was collected by filtration under reduced pressure and washed thoroughly with water and Et_2_O to afford chromatographically pure green crystals. Yield: 93%. M.p. > 300 °C. UV/Vis [MeOH/CH_2_Cl_2_, 99/1 (*v*/*v*)] λ_max_ (log ε): 660 (4.85); ¹H-NMR (DMSO-*d*_6_) δ: 8.22 (2H, s, Ar*H*), 7.72 (2H, d, *J* = 8.0, Ar*H*), 7.32 (2H, d, *J* = 8.0, Ar*H*), 5.78 (2H, s, C*H*=C), 4.25 (4H, d, *J* = 6.8, NC*H*_2_CH_3_), 1.35–1.30 (6H, m, NCH_2_C*H*_3_). HRMS (ESI-TOF) *m/z*: 683.88936 ([M − I]^+^, calc. for C_24_H_20_I_2_N_3_O_2_S_2_: 683.88761).

#### 3.1.4. Synthesis of 3-Ethyl-2-[3-(3-ethyl-6-iodo-3*H*-benzothiazol-2-ylidenemethyl)-2-methoxy-4-oxo-cyclobut-2-enylidenemethyl]-6-iodobenzothiazol-3-ium trifluoromethanesulfonate (**4**)

To a solution of squaraine dye **3** (0.75 g, 1.10 mmol) in anhydrous CH_2_Cl_2_ (250 mL), stirred under N_2_ atmosphere at r.t., was added an excess of CF_3_SO_3_CH_3_ (1.0 mL, 8.80 mmol). After 22 h the reaction mixture was quenched with 5% (*m*/*v*) aqueous NaHCO_3_ and the organic layer, after being separated by decantation, was dried over anhydrous Na_2_SO_4_. The solvent was then removed under reduced pressure to afford green crystals, which were found by TLC to have sufficient purity to be used in the next step without further purification. Yield: 89.6%. M.p. > 300 °C; UV/Vis [MeOH/CH_2_Cl_2_, 99/1 (*v*/*v*)] λ_max_ (log ε): 644 (5.34).

#### 3.1.5. Synthesis of 2-[2-Amino-3-(3-ethyl-6-iodo-3*H*-benzothiazol-2-ylidenemethyl)-4-oxocyclobut-2-enylidenemethyl]-3-ethyl-6-iodobenzothiazol-3-ium iodide (**5a**)

To a solution of *O*-methylsquaraine **4** (0.30 g, 0.35 mmol) in anhydrous CH_2_Cl_2_ (125 mL), vigorously stirred under N_2_ atmosphere at r.t., was added stepwise a 15.5-fold excess of a solution of 2 M NH_3_ in MeOH. The reaction mixture was stirred at r.t. for 7 days. The resulting precipitate was collected by filtration under reduced pressure, washed with water and Et_2_O and dissolved in MeOH. To this solution was added an approximately equal volume of 14% (*m*/*v*) aqueous KI. After stirring at r.t. for 1h, the precipitated dye was collected by filtration under reduced pressure and washed with water and Et_2_O. Yield: 84%. Purple crystals. M.p. > 300 °C. UV/Vis [MeOH/CH_2_Cl_2_, 99/1 (*v*/*v*)] λ_max_ (log ε): 661 (5.40). ^1^H-NMR (DMSO-*d*_6_) δ: 8.53 (2H, s, N*H*_2_), 8.34 (2H, s, Ar*H*), 7.82 (2H, d, *J* = 8.0, Ar*H*), 7.46 (2H, d, *J* = 8.6, Ar*H*), 6.16 (2H, s, C*H*=C), 4.27 (4H, br q, *J* = 6.8, NC*H*_2_CH_3_), 1.35 (6H, br t, *J* = 6.6, NCH_2_C*H*_3_). ^13^C-NMR (DMSO-*d*_6_) δ: 173.5, 165.7, 159.8, 157.5, 140.0, 136.0, 130.5, 129.7, 114.5, 88.0, 86.1, 41.3, 12.0. HRMS (ESI-TOF) *m/z*: 683.91317 ([M − I]^+^, calc. for C_24_H_20_I_2_N_3_OS_2_: 683.91168).

#### 3.1.6. Synthesis of 3-Ethyl-2-[3-(3-ethyl-6-iodo-3*H*-benzothiazol-2-ylidenemethyl)-2-(methylamino)-4-oxocyclobut-2-enylidenemethyl]-6-iodobenzothiazol-3-ium iodide (**5b**)

A mixture of *O*-methylsquaraine **4** (0.10 g, 0.12 mmol) and an excess of 2 M methylamine in MeOH (0.047 mL, 1.20 mmol), in anhydrous CH_2_Cl_2_ (125 mL), was stirred at r.t. under N_2_ for about 2 days. The reaction mixture was then worked-up as above for **5a**. Yield: 70%. Purple crystals. M.p. > 300 °C. UV/Vis [MeOH/CH_2_Cl_2_, 99/1 (*v*/*v*)] λ_max_ (log ε): 671 (4.92). ^1^H-NMR (DMSO-*d*_6_) δ: 8.76 (1H, s, N*H*CH_3_), 8.41 (1H, s, Ar*H*), 8.35 (1H, s, Ar*H*), 7.85–7.80 (2H, m, Ar*H*), 7.52 (1H, d, *J* = 8.6, Ar*H*), 7.46 (1H, d, *J* = 8.6, Ar*H*), 6.17 (1H, s, C*H*=C), 6.05 (1H, s, C*H*=C), 4.38 (2H, br q, *J* = 7.3, NC*H*_2_CH_3_), 4.28 (2H, br q, *J* = 7.0, NC*H*_2_CH_3_), ~3.30 (NHC*H*_3_, overlapped with residual HOD signal), 1.32–1.26 (6H, m, NCH_2_C*H*_3_). ^13^C-NMR (DMSO-*d_6_*): 173.2, 164.1, 160.4, 159.0, 157.0, 156.4, 140.1, 140.0, 136.3, 136.0, 130.8, 130.5, 130.1, 130.0, 114.9, 114.6, 88.9, 88.3, 86.8, 86.2, 41.7, 41.1, 30.4, 12.3, 12.2. HRMS (ESI-TOF) *m/z*: 697.92882 ([M − I]^+^, calc. for C_25_H_22_I_2_N_3_OS_2_: 697.92727).

#### 3.1.7. Synthesis of 3-Ethyl-2-{3-(3-ethyl-6-iodo-3H-benzothiazol-2-ylidenemethyl)-2-[bis(2-hydroxy-ethyl)amino]-4-oxocyclobut-2-enylidenemethyl}-6-iodobenzothiazol-3-ium iodide (**5c**)

To a solution of *O*-methylsquaraine **4** (0.30 g, 0.35 mmol) in anhydrous CH_2_Cl_2_ (125 mL), under N_2_ atmosphere, was added excess diethanolamine (0.084 mL, 0.89 mmol). After being stirred at r.t. for 9 days the reaction mixture was worked-up as above for **5a** and the resulting solid recrystallized from MeOH. Yield: 74%. Purple crystals. M.p. > 300 °C. UV/Vis [MeOH/CH_2_Cl_2_, 99/1 (*v*/*v*)] λ_max_ (log ε): 679 (4.91). ^1^H-NMR (DMSO-*d*_6_): 8.39 (2H, s, Ar*H*), 7.83 (2H, d, *J* = 8.4, Ar*H*), 7.50 (2H, d, *J* = 8.6, Ar*H*), 6.09 (2H, s, C*H*=C), 5.17 (2H, s, NCH_2_CH_2_O*H*), 4.37 (4H, br s, NC*H*_2_CH_3_), 3.78 (8H, br s, NC*H*_2_C*H*_2_OH), 1.28 (6H, br s, NCH_2_C*H*_3_). ^13^C-NMR (DMSO-*d_6_*): 173.5, 164.0, 159.2, 156.0, 140.0, 136.19, 130.6, 130.2, 114.8, 88.6, 87.9, 59.2, 53.7, 41.4 12.1. HRMS (ESI-TOF) *m/z*: 771.96560 ([M − I]^+^, calc. for C_28_H_28_I_2_N_3_O_3_S_2_: 771.96288).

#### 3.1.8. Synthesis of 3-Ethyl-2-{3-(3-ethyl-6-iodo-3*H*-benzothiazol-2-ylidenemethyl)-2-[(2-hydroxy-ethyl)amino]-4-oxocyclobut-2-enylidenemethyl}-6-iodobenzothiazol-3-ium iodide (**5d**)

To a solution of *O*-methylsquaraine **4** (0.11 g, 0.13 mmol) in anhydrous CH_2_Cl_2_ (125 mL) and CH_3_CN (60 mL), under N_2_ atmosphere, was added excess ethanolamine (0.38 mL, 0.63 mmol). The reaction mixture was stirred at r.t. for about 5 days. The reaction mixture was worked-up as above for **5a**. Yield: 52%. Purplish blue crystals. M.p. > 300 °C. UV/Vis [MeOH/CH_2_Cl_2_, 99/1 (*v*/*v*)] λ_max_ (log ε): 670 (4.96). ^1^H-NMR (DMSO-*d*_6_): 8.90 (1H, s, N*H*CH_2_CH_2_OH), 8.39 (1H, s, Ar*H*), 8.33 (1H, s, Ar*H*) 7.84–7.78 (2H, m, Ar*H*), 7.52–7.43 (2H, m, Ar*H*), 6.29 (1H, s, C*H*=C), 5.95 (1H, s, C*H*=C), 5.13 (1H, s, NHCH_2_CH_2_O*H*), 4.35–4.28 (4H, m, NHC*H*_2_CH_3_), 3.74 (4H, br s, NHC*H*_2_C*H*_2_OH), 1.34–1.25 (6H, m, NCH_2_C*H*_3_). ^13^C-NMR (DMSO-*d*_6_): 173.6, 163.9, 160.4, 158.9, 157.3, 156.0, 140.1, 140.0, 136.3, 136.1, 130.8, 130.5, 130.1, 114.9, 114.6, 88.9, 88.2, 87.2, 86.5, 60.5, 46.5, 41.7, 41.1, 12.4, 12.0. HRMS (ESI-TOF) *m/z*: 727.93938 ([M − I]^+^, calc. for C_26_H_24_I_2_N_3_O_2_S_2_: 727.93762).

#### 3.1.9. Synthesis of 3-Ethyl-2-{3-(3-ethyl-6-iodo-3*H*-benzothiazol-2-ylidenemethyl)-2-[(2-hydroxy-ethyl)methylamino]-4-oxocyclobut-2-enylidenemethyl}-6-iodobenzothiazol-3-ium iodide (**5e**)

A mixture of *O*-methylsquaraine **4** (0.99 g, 0.12 mmol) and *N*-methylethanolamine (0.14 mL, 1.78 mmol) in anhydrous CH_2_Cl_2_ (125 mL) and CH_3_CN (45 mL) was stirred at r.t., under N_2_ atmosphere, for approximately 5 days. The precipitated solid was collected by filtration under reduced pressure and washed with water and Et_2_O. The mother liquor was washed with water and the organic layer, after being separated by decantation, was dried over anhydrous Na_2_SO_4_ and evaporated to dryness under reduced pressure. The combined solids underwent counter ion exchange as described for **5a**. Yield: 56%. Blue crystals. M.p. > 300 °C. UV/Vis [MeOH/CH_2_Cl_2_, 99/1 (*v*/*v*)] λ_max_ (log ε): 682 (4.43). ^1^H-NMR (DMSO-*d*_6_): 8.39 (2H, s, Ar*H*), 7.83 (2H, d, *J* = 8.6, Ar*H*), 7.50 (2H, d, *J* = 8.8, Ar*H*), 6.10 (2H, s, C*H*=C), 5.16 (1H, s, NCH_3_CH_2_CH_2_O*H*), 4.39 (4H, s, NC*H*_2_CH_3_), 3.74 (4H, s, NCH_3_C*H*_2_C*H*_2_OH), 3.48 (3H, s, NC*H*_3_CH_2_CH_2_OH), 1.28 (6H, t, *J* = 5.5, NCH_2_C*H*_3_). HRMS (ESI-TOF) *m/z*: 741.95503 ([M − I]^+^, calc. for C_27_H_26_I_2_N_3_O_2_S_2_: 741.95297).

### 3.2. Photochemistry

#### Singlet Oxygen Quantum Yield Determinations

The singlet oxygen measurement set-up was assembled in our laboratory as previously described [43]. As an excitation source we used a nitrogen laser, excitation wavelength = 337 nm. The detector was an InGaAs CCD (model i-Dus from Andor Technology Limited, Belfast, UK) working at low temperature (−60 °C) coupled to a fixed spectrograph, model Shamrock 163i, also from Andor. Phenazine (standard) was used at O.D. = 0.6 in chloroform. The Φ_Δ_ values were obtained by comparing the total area of the emission spectra for the reference and for each dye under study in the same solvent, with the same optical density at the excitation wavelength. Phenazine (standard) was used at OD = 0.60 in CHCl_3_.

### 3.3. Biology

#### 3.3.1. General

Fetal bovine serum (FBS), l-glutamine, Hank’s balanced salt solution (HBSS), trypsin-EDTA (ethylenediaminetetraacetic acid), penincillin/streptomycin solution (100 U/mL and 100 mg/mL, respectively), and RPMI-1640 were from Hyclone (Logan, UT, USA). Acetic acid, ellipticine, sulforhodamine B (SRB), trypan blue, trichloroacetic acid (TCA) and Tris were purchased from Sigma Chemical Co. (St. Louis, MO, USA). Water was treated in a Milli-Q water purification system (TGI Pure Water Systems, Greenville, SC, USA).

#### 3.3.2. Preparation of the Solutions of Aminosquaraine Dyes

Stock solutions of the aminosquaraines **5a**–**e** with different concentrations (0.0375 μM, 0.15 μM, 0.6 μM, 6 μM, 12 μM, 25 μM, 50 μM, 500 μM) were prepared using a solution of 3% DMSO in DMEM and kept at−20 °C. Prior to the in vitro assays diluted solutions were prepared with the same solvent to obtain solutions with final concentrations of 0.03 μM, 0.3 μM, 0.6 μM, 1.25 μM, 2.5 μM and 25 μM. For the HeLa MCF-7 cell lines solutions with final concentrations of 0.0075 μM and 0.001875 μM were additionally prepared once the inhibition percentage for the 0.03 μM solution was already superior to 50%.

#### 3.3.3. Photodynamic Treatment

The cytotoxicity of the squaraine dyes was tested in the dark and under irradiation. For irradiation of the cells a halogen/tungsten lamp (24 V and 250 W, Osram, Carnaxide, Portugal) was used with a fluence rate of 23–24 µW/cm^2^ (measured with an ILT 1400-A radiometer equipped with a SEL033 detector, ILT, Peabody, MA, USA). The cells were irradiated continuously for 30 min. A 3% aqueous solution of K_2_Cr_2_O_7_ was placed between the lamp and the cells as a liquid cut off filter to remove light of wavelength shorter than ~500 nm. The temperature to which the cells were exposed was carefully monitored to guarantee cell viability.

#### 3.3.4. Evaluation of Cytotoxicity in Human Tumor Cell Lines

Four human tumor cell lines were used: MCF-7 (breast adenocarcinoma), NCI-H460 (non-small cell lung carcinoma), HeLa (cervical carcinoma) and HepG2 (hepatocellular carcinoma) from DSMZ (Leibniz-Institut DSMZ-Deutsche Sammlung von Mikroorganismen und Zellkulturen GmbH, Mönchengladbach, Germany). Cells were routinely maintained as adherent cell cultures in RPMI-1640 medium containing 10% heat-inactivated FBS and 2 mM glutamine at 3 °C, in a humidified air incubator containing 5% CO_2_. Each cell line was plated at an appropriate density (7.5 × 10^3^ cells/well for MCF-7 and NCI-H460, and 1.0 × 10^4^ cells/well for HeLa and HepG2) in 96-well plates, allowed to attach for 24 h and then treated with different concentrations of each squaraine dye. Controls were set with the cells in the absence of the dyes in 3% DMSO in DMEM and in the growth medium only. After the incubation step (24 h), the cells were submitted to the photodynamic treatment for 30 min. Controls in the absence of light were performed on every test. Neither DMSO alone, nor light alone, nor the combination of both induced toxicity on the cells. Then, the growth medium was changed and the cells were incubated for further 24 h. Following this incubation period, the adherent cells were fixed by adding cold 10% TCA (100 µL) and incubated for 60 min at 4 °C. Plates were then washed with deionized water and subsequently dried. SRB solution (0.1% in 1% acetic acid, 100 µL) was then added to each plate well and incubated for 30 min at room temperature. Unbound SRB was removed by washing with 1% acetic acid. Plates were air dried, the bound SRB was solubilized with 10 mM Tris (200 µL) and the absorbance was measured at 540 nm in an ELX800 Microplate Reader (Bio-Tek Instruments Inc., Winooski, VT, USA) [46]. The results were expressed in GI_50_ values, which correspond to the concentration of dye that causes 50% inhibition of cell growth. All the assays were performed in duplicate. Ellipticine was used as standard.

#### 3.3.5. Evaluation of Cytotoxicity in a Porcine Liver Primary Cell Culture

A cell culture was prepared from a freshly harvested porcine liver obtained from a local slaughter house, and it was designed as PLP2. Briefly, the liver tissues were rinsed in Hank’s balanced salt solution containing 100 U/mL penicillin and 100 µg/mL streptomycin and divided into 1×1 mm^3^ explants to ensure that all liver cells were able to access the nutrients provided by the medium. Some of these explants were placed in 25 cm^2^ tissue flasks in DMEM medium supplemented with 10% fetal bovine serum, 2 mM non-essential amino acids and 100 U/mL penicillin, 100 mg/mL streptomycin and incubated at 37 °C with a humidified atmosphere containing 5% CO_2_. The medium was changed every two days. Cultivation of the cells was continued with direct monitoring every two or three days using a phase contrast microscope. Before confluence was reached, cells were subcultured and plated in 96-well plates at a density of 1.0 × 10^4^ cells/well, and cultivated in DMEM medium with 10% FBS, 100 U/mL penicillin and 100 µg/mL streptomycin [47]. The PLP2 cells were incubated with the squaraine dyes and submitted to the two independent processes—dark and irradiation—as described earlier for the human tumor cell lines. The SRB assay was performed according to the procedure previously described. The results were expressed in GI_50_ values. All the assays were performed in duplicate. Ellipticine was used as standard.

#### 3.3.6. Statistical Analysis

All the assays were carried out in duplicate. The results are expressed as mean values and standard deviation. The results were analysed using a Student´s *t*-test to determine the significant difference among the different samples, with α = 0.05. This treatment was carried out using SPSS v. 22.0 program (IBM Corp., Armonk, NY, USA).

## 4. Conclusions

In conclusion, the synthesized aminosquaraine dyes, with strong absorption within the phototherapeutic window, showed photodynamic activity against the human tumor cell lines studied (MCF-7, NCI-H460, HeLa, and HepG2), inhibiting cell growth upon irradiation more than in the absence of light, in some cases substantially. When compared to the non-halogenated analogues dyes **5a**–**e** exhibited, in general, lower GI_50_ values under irradiation, which is probably related to the enhancement of singlet oxygen production through the so-called heavy atom effect. The difference of cytotoxicity observed in the dark and upon irradiation, turns some of the dyes potential candidates as photosensitizers for PDT, in particular compound **5a**, which showed the lowest toxicity for the non-tumor primary PLP2 cells.

## Figures and Tables

**Table 1:** Visible spectral data and fluorescence and singlet oxygen generation quantum yields of squaraine dyes **3** and **5a**–**e**.

Dye	R_1_	R_2_	λ_max_^a^ (nm)	log ε_max_^a^	Φ_F_^b^	Φ_∆_^b^
**3**	-	-	660	4.85	0.03	0.05
**5a**	H	H	661	5.40	0.02	0.22
**5b**	H	CH_3_	670	4.96	0.03	0.26
**5c**	CH_2_CH_2_OH	CH_2_CH_2_OH	679	4.91	0.01	0.09
**5d**	H	CH_2_CH_2_OH	671	5.04	0.11	0.19
**5e**	CH_3_	CH_2_CH_2_OH	682	4.50	0.09	0.16

^a^ In MeOH/CH_2_Cl_2_, 99/1 (*v*/*v*); ^b^ In CHCl_3_.

**Table 2:** Cytotoxicity (GI_50_ values, μM) of aminosquaraine dyes **5a**–**e** (mean ± SD).

Dye	Condition	HeLa	HepG2	MCF-7	NCI-H460	PLP2
**5a**	Dark	2.54 ± 0.02	20 ± 2	15 ± 1	15.3 ± 0.8	17.5 ± 0.1
	Irradiated	0.004 ± 0.0001	0.08 ± 0.001	0.01 ± 0.001	0.19 ± 0.02	2.0 ± 0.1
	*t*-Student test *p*-value	<0.001	<0.001	<0.001	<0.001	<0.001
**5b**	Dark	1.2 ± 0.1	15 ± 2	10.0 ± 0.7	8.1 ± 0.9	5.7 ± 0.6
	Irradiated	0.011 ± 0.01	0.06 ± 0.001	0.01 ± 0.001	0.18 ± 0.07	0.03 ± 0.001
	*t*-Student test *p*-value	<0.001	<0.001	<0.001	<0.001	<0.001
**5c**	Dark	2.0 ± 0.1	23 ± 2	>25	23 ± 2	22.0 ± 0.9
	Irradiated	0.31 ± 0.03	0.26 ± 0.03	0.42 ± 0.06	0.98 ± 0.04	0.44 ± 0.01
	*t*-Student test *p*-value	<0.001	<0.001	-	<0.001	<0.001
**5d**	Dark	1.8 ± 0.1	>25	>25	>25	>25
	Irradiated	0.19 ± 0.01	0.38 ± 0.03	0.25 ± 0.04	0.89 ± 0.01	0.98 ± 0.02
	*t*-Student test *p*-value	<0.001	-	-	-	-
**5e**	Dark	2.1 ± 0.2	11.4 ± 0.9	9.6 ± 0.2	13 ± 1	11.9 ± 0.2
	Irradiated	0.19 ± 0.02	0.19 ± 0.01	0.23 ± 0.02	0.24 ± 0.03	0.42 ± 0.01
	*t*-Student test *p*-value	<0.001	<0.001	<0.001	<0.001	<0.001
